# Integrating Transcriptomics and Proteomics to Characterize the Intestinal Responses to Cadmium Exposure Using a Piglet Model

**DOI:** 10.3390/ijms25126474

**Published:** 2024-06-12

**Authors:** Yikun Li, Yiling Pan, Yulong Yin, Ruilin Huang

**Affiliations:** 1 College of Animal Science, South China Agricultural University, Guangzhou 510642, China; liyikun17@mails.ucas.edu.cn; 2 Institute of Subtropical Agriculture, Chinese Academy of Sciences, Changsha 410125, China

**Keywords:** cadmium exposure, intestinal responses, transcriptomics, proteomics, piglets

## Abstract

Cadmium (Cd) is a heavy metal element with a wide range of hazards and severe biotoxicity. Since Cd can be easily accumulated in the edible parts of plants, the exposure of humans to Cd is mainly through the intake of Cd-contaminated food. However, the intestinal responses to Cd exposure are not completely characterized. Herein, we simulated laboratory and environmental Cd exposure by feeding the piglets with CdCl_2_-added rice and Cd-contaminated rice (Cdcr) contained diet, as piglets show anatomical and physiological similarities to humans. Subsequent analysis of the metal element concentrations showed that exposure to the two types of Cd significantly increased Cd levels in piglets. After verifying the expression of major Cd transporters by Western blots, multi-omics further expanded the possible transporters of Cd and found Cd exposure causes wide alterations in the metabolism of piglets. Of significance, CdCl_2_ and Cdcr exhibited different body distribution and metabolic rewiring, and Cdcr had stronger carcinogenic and diabetes-inducing potential. Together, our results indicate that CdCl_2_ had a significant difference compared with Cdcr, which has important implications for a more intense study of Cd toxicity.

## 1. Introduction

Cadmium (Cd) is a toxic heavy metal element, and about 30,000 tons of Cd are released into the environment every year, most of which are strongly associated with human activities. Cd in agricultural soil has the characteristics of strong toxicity and non-biodegradability, but it is more easily enriched by plants, including rice, wheat, potatoes, and other staple grains [1]. Cd tends to accumulate in edible parts of crops; however, most crops contaminated by Cd do not exhibit negative effects on growth [2], which makes crops contaminated by Cd difficult to identify. Therefore, in the non-smoking population, approximately 90% of Cd intake is originally from the diet, among which rice is the uppermost contributor to dietary Cd intake [3]. Cd intake at concentrations well above the permissible limits in food products could result in liver and kidney injury, osteotoxicity, cancer, etc., seriously affecting human food security [4,5,6,7]. Dietary Cd exposure damages the intestine once it wastrapped on the epithelium surface [8,9], after which it preferentially accumulates and is absorbed in the small intestine before transferring into the blood [10]. Studies with mice, rats, chickens, and pigs have shown that the duodenum has the highest Cd absorption rate [10,11,12,13]. Cd is a toxic non-essential mineral element, and there are no Cd-specific ion channels or transporters on the enterocytes [14].

As such, current studies demonstrate that Cd is absorbed by mimicking the other bivalent metal elements and utilizing their transporters, like iron (Fe), zinc (Zn), and calcium (Ca) [15,16,17,18,19]. After Cd enters the cell, Cd binds to specific proteins, such as metallothionein (MT), which is an intracellular metal-binding and regulator protein with an important cytoprotective function [20]. Notably, MT also serves as a carrier protein for circulating Cd, which combines Cd to form MT-Cd complexes and then exits from enterocytes and enters into the systemic circulation [20]. At present, a large number of studies have shown the cytotoxicity of Cd. Studies have shown that Cd exposure causes mitochondrial disorders and endoplasmic reticulum stress, leading to cell necrosis or apoptosis [21,22]. Cd also interferes with the electron transport chain; increases oxidative stress levels and intracellular ROS content; induces the oxidative damage of DNA, lipids, and proteins; and induces cell death [23]. In addition, Cd also promotes the expression of cancer-promoting genes in some cells and activates cellular protein kinases and DNA methylation, thereby triggering the occurrence and progression of tumors [24].

Remarkably, among these studies, quite a few have reported the adverse effects caused by the laboratory simulation of Cd exposure (CdCl_2_). However, natural Cd contaminants, such as Cd-contaminated rice, contain a diverse oxidation state with various chemical compounds [25], most of which is in the form of Cd–thiolate, and the remainder of the Cd (8–34%) is associated with carboxyl compounds, histidine, or phytate [25]. In addition, Cd can bind to many organic ligands, electron donors, and proteins in cereals, with possibly hundreds of types being involved [26], which makes it difficult to ascertain how Cdis transported and the toxicity effects of Cd. To date, how the Cd is transported through the intestine into the body has not been fully clarified. Meanwhile, research on the process of Cd toxicity in humans is basically conducted using non-invasive samples (like blood, urine, saliva, etc.), while research on metabolic changes in the body mainly relies on the use of rodents [27]. As a model animal, research has widely proven that pigs are more genetically similar to humans than mice and are cheaper and more accessible than monkeys [28]. Pig intestines are also similar to human intestines, and piglet gut models have been established to mimic the responses of humans to foreign substances as well as interventions through nutrients [29].

Therefore, in this study we use rice contaminated with CdCl_2_ (CdCl_2_) and naturally grown Cd-contaminated rice (Cdcr) to comprehensively and systematically explore the similarities and differences in the intestinal responses to Cd exposure of piglets via performing multi-omics profiling. We provide differences between CdCr and CdCl2 in many aspects such as transporters, tissue distribution, potential toxicity, etc. Our findings indicate that CdCl2 may not completely represent natural Cd contaminants, which has implications for the comprehension of human Cd toxicity.

## 2. Results

### 2.1. Establishment of Cd-Exposed Piglet Model

**Figure 1:**
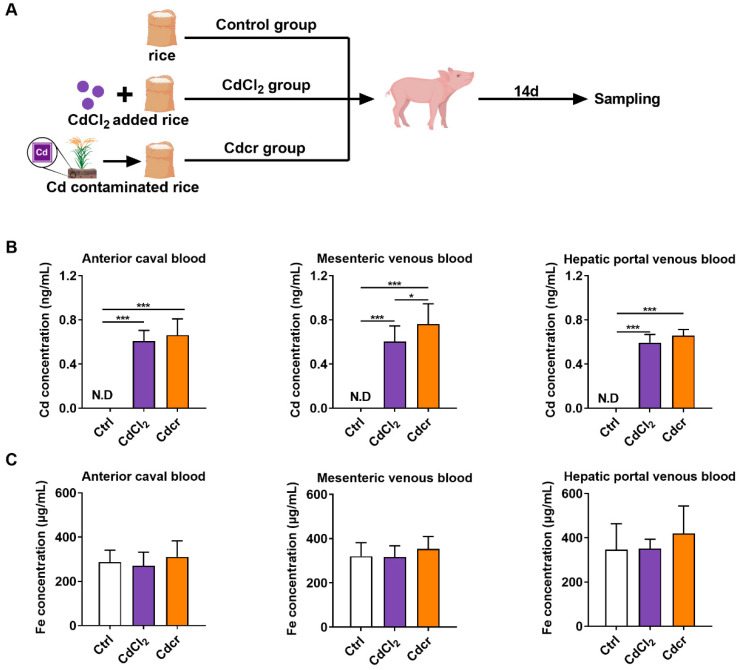
Establishment of Cd-exposed piglet model. (**A**) The diagram shows experimental procedures. (**B**) Cd concentration in piglet blood after exposure to CdCl_2_ and Cdcr diets, n = 8. (**C**) Fe concentration in piglet blood after exposure to CdCl_2_ and Cdcr diets, n = 8. Data were analyzed by unpaired *t*-test and represented as means ± SD. * *p* < 0.05 and *** *p* < 0.001.

After a 14-day experimental period (Figure 1A), Cd content in the blood (including anterior caval blood, mesenteric venous blood, and hepatic portal venous blood) of piglets in the CdCl_2_ and Cdcr group was significantly increased (Figure 1B), confirming that Cd is effectively ingested into piglets. Interestingly, although the uncontaminated rice contained a small amount of Cd, no Cd was detected in the blood of piglets in the uncontaminated rice contained diet (Ctrl) group (Figure 1B). It has been reported that Cd exposure competes with Fe transport into the intestines [30], potentially resulting in decreased Fe absorption and Fe deficiency anemia, affecting the growth of piglets [31]. However, in our results, the blood Fe concentration of piglets fed with Cd was comparable to those in the Ctrl group, showing that Cd exposure in our experiment might not affect the Fe levels (Figure 1C), which may be related to the short trial period and adequate iron reserves in the piglets.

### 2.2. Cd Deposition in the Organs of Piglets after Feeding Cd-Containing Diets

**Figure 2:**
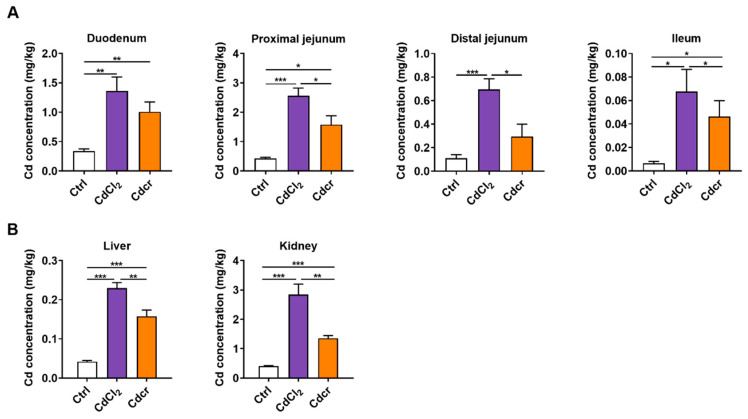
Cd content in piglet organs after Cd exposure. (**A**) Cd content in different segments of the small intestine after exposure to CdCl_2_ and Cdcr diets to piglets, n = 8. (**B**) Cd content in piglet liver and kidney after exposure to CdCl2 and Cdcr diets. n = 8. Data were analyzed by unpaired *t*-test and represented as means ± SD. * *p* < 0.05, ** *p* < 0.01 and *** *p* < 0.001.

In our results, the Cd content was significantly increased in all segments of the small intestine in both Cd diet groups (Figure 2A), especially in the duodenum and proximal jejunum (Figure 2A), which is congruent with the data previously described in the literature [12,32,33]. Furthermore, the intestinal Cd content of piglets in the CdCl_2_ group was statistically higher than those in the Cdcr group, except for the duodenum (Figure 2A). Likewise, Cd was also accumulated in the liver and kidney, as those are the main target organs for Cd (Figure 2B). It seems CdCl_2_ had higher bioavailability, accumulating more Cd in the intestine, liver and kidney (Figure 2A,B), but the CdCl_2_ group exhibited lower blood Cd content and higher organ deposition than the CdCr group (Figure 1B and Figure 2B). Interestingly, we found that the pH of duodenal contents remarkably decreased after Cd exposure (Figure S1A), and the gastrointestinal pH affects Cd transportation [34]. Together, pH may promote the CdCl2 group to have higher Cd content in the duodenum.

In addition, Cd exposure also affects the deposition of other metal elements [35]. In our results, dietary Cd exposure had the greatest impact on Zn deposition in the liver and kidneys, but not Fe, Cu, and Mn, and the amount of Zn deposition in the Cdcr group was lower than that in the CdCl2 group. The absorption of Cd causes alterations in the contents of other metal elements simultaneously, and it has been reported that Zn might compete for Cd uptaking and culminate in reducing Cd toxicity [36]. Accordingly, in the Cdcr group, the Zn content in the liver and kidneys was reduced after Cd exposure only (Figure S1B,C), while Cu and Mn in the kidneys were lower than that in the CdCl_2_ group (Figure S1C). CdCl2 exposure had no impact on the level of Fe, Cu, Zn, and Mn (Figure S1B,C).

### 2.3. Expressions of Cd-Related Transporters in the Gut of Cd-Exposed Piglets

**Figure 3:**
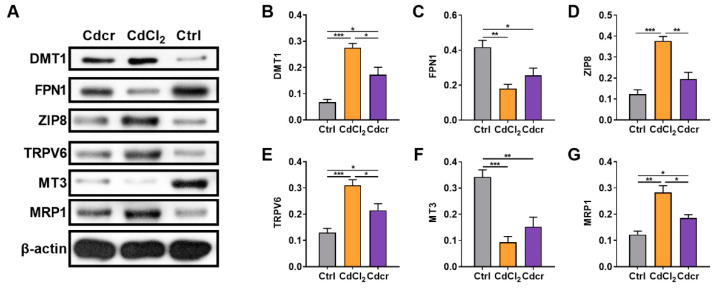
Expression of transporters associated with Cd transport in the duodenum. (**A**) Protein expression of DMT1 (**B**), ferroportin 1 (FPN1) (**C**), Zrt-/Irt-like protein 8 (ZIP8/ SLC39A8) (**D**), transient receptor potential cation channel subfamily V member 6 (TRPV6) (**E**), metallothionein-3 (MT3) (**F**), multidrug resistance protein 1 (MRP1) (**G**) in the duodenum of Cd exposed piglets (n = 4). Data were analyzed by unpaired *t*-test and represented as means ± SD. * *p* < 0.05, ** *p* < 0.01 and *** *p* < 0.001.

As a non-essential mineral element, there is no specific Cd ion channel or transporter in the body [14]. Therefore, we detected several reported Cd-relevant transporters in different segments of the small intestine in each group (Figure 3A and Figure S2A,H,O). DMT1 (uptake) and ferroportin 1 (FPN1) (efflux) are the critical Fe transporters in the intestine that participate in Cd transportation [16,33,37]. Notably, previous studies have reported that the Cd treatment increases the expression of FPN1 governed by Metal Transcription Factor-1 [38], but reduced expression of FPN1 after Cd exposure might be related to anemia [39]. Predictably, in the CdCl_2_ and Cdcr groups, the protein expression of DMT1 was highly increased in all parts of the small intestine (Figure 3B and Figure S2B,I,P), while FPN1 protein expression in two Cd treatment groups was all lower than in the Ctrl group (Figure 3C and Figure S2C,J,Q). Furthermore, the protein expression of DMT1 was higher in the duodenum (Figure S2C), distal jejunum (Figure S2I), and ileum (Figure S2Q) of CdCl_2_-fed piglets compared to those in the Cdcr group. The protein expression of FPN1 in the proximal jejunum (Figure S2C) of piglets in the CdCl_2_ group was higher but then lower in the distal jejunum and ileum (Figure S2J,Q). The ZIP family members (ZIPs), such as ZIP8 (also known as SLC39A8), have been shown to mediate Cd uptake in different cell types [40,41]. Our results confirmed that the expression of ZIP8 was increased to varying degrees in the two treatment groups (Figure 3D and Figure S2D,K,R). Moreover, in the small intestine, except for the proximal jejunum, the expression of ZIP8 in the CdCl_2_ group was higher than that in the Cdcr group (Figure 3D and Figure S2K,R). Transient receptor potential cation channel subfamily V member 6 (TRPV6) is a critical Ca transporter in epithelial tissues which has also been reported to transport various heavy metals, including Cd [42,43]. The protein expression of TRPV6 was substantially increased, chiefly in the CdCl_2_ group (Figure 3E and Figure S2E,L,S).

Next, we detected the expression of several proteins participating in the Cd efflux and detoxification. MT is a metal-binding protein that defends against heavy metals by chelating metal elements, protecting cells from acute metal exposure [20,44]. Intriguingly, we found that Cd exposure in piglets dramatically reduced the expression of metallothionein-3 (MT3) (Figure 3F and Figure S2F,M,U). However, Cd exposure in piglets induced the expression of multidrug resistance protein 1 (MRP1) (Figure 3G and Figure S2G,N,V), a protein that generally excretes Cd out of the cell to exert the detoxification function [45]. Seemingly, the intrinsic capacity of cells to alleviate the Cd-induced cytotoxicity may not entirely rely on MT-mediated chelation, and the efflux proteins, such as MRP1, may have a more important role [46].

### 2.4. Transcriptomic Analysis on Responses of the Duodenum of Piglets Fed Cd Containing Diets

**Figure 4:**
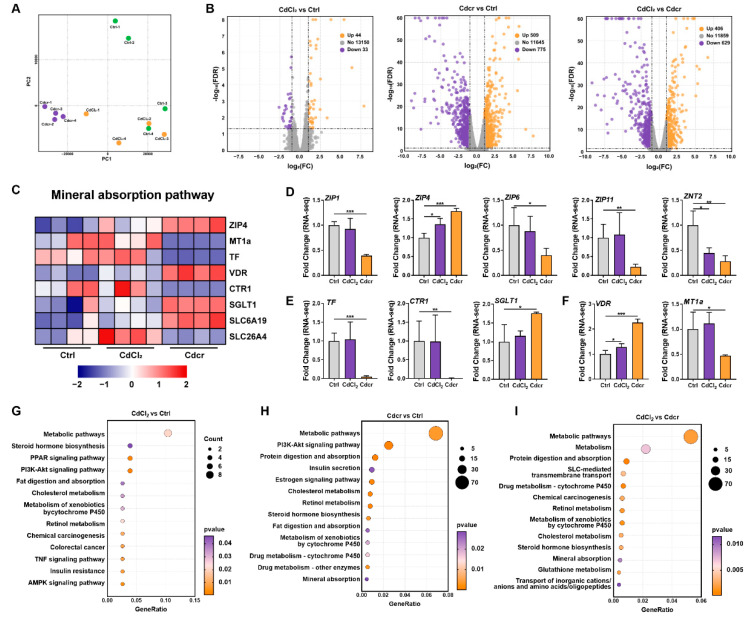
Transcriptomic Profiles after piglets were fed with CdCl_2_ and Cdcr contained diets. (**A**) Principal component analysis (PCA) was used to evaluate the reproducibility of each set of samples (n = 4). (**B**) Volcano plots showed DEGs and up- and down-regulation between groups. (**C**) Heatmap demonstrated DEGs in mineral absorption pathway. The expression of DEGs related to ZIP family (**D**), Cd transport (**E**) and intracellular sensing and chelation (**F**) (n = 4) after piglets were fed with CdCl2 and Cdcr contained diets. (**G**–**I**) Metabolism-related KEGG pathways enriched by DEGs (top 13). Data were analyzed by unpaired *t*-test and represented as means ± SD. * *p* < 0.05, ** *p* < 0.01 and *** *p* < 0.001.

To systematically decipher the underlying biological significance and difference between the two Cd exposures, a transcriptome (RNA-seq) profiling approach was applied to the three groups (Ctrl, CdCl_2_, and Cdcr) (Figure 4A). A total of 13,357 genes were quantified in the whole intestinal samples, and 11,744 protein-coding genes were preserved after filtering using the Ensembl database. We used edgeR for differential analysis with FC ≥ 2 and FDR ≤ 0.01 as screening criteria. Based on these criteria, as the volcano plot showed, 44 genes were up-regulated and 33 genes were down-regulated in the CdCl_2_ vs. Ctrl group, 509 genes were up-regulated and 775 genes were down-regulated in the Cdcr vs. Ctrl group, and 406 genes were up-regulated and 629 genes were down-regulated in the CdCl_2_ vs. Cdcr group (Figure 4B).

**Figure 5:**
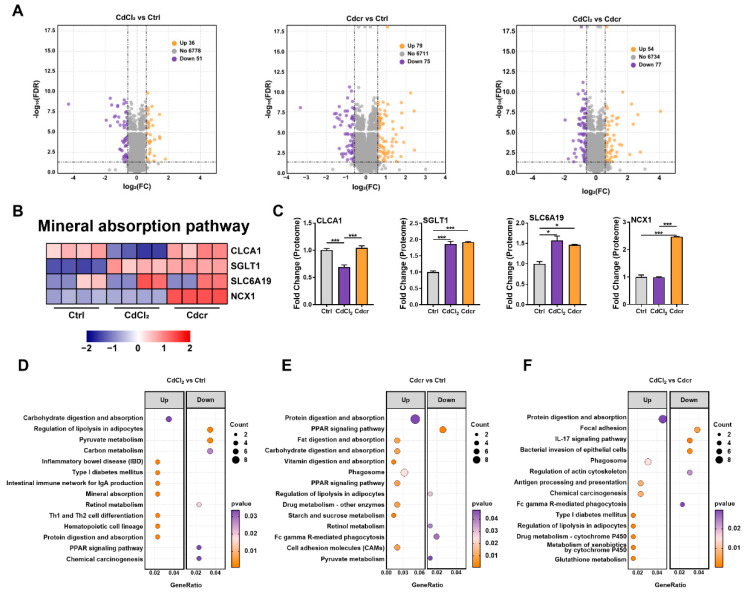
Proteome Profiles after piglets were fed with CdCl_2_ and Cdcr contained diet. (**A**) Volcano plots showed DEPs and up- and down-regulation between groups. (**B**) Heatmap demonstrated DEPs in mineral absorption pathway. (**C**) DEPs related to Cd transport (n = 4). (**D**–**F**) Metabolism-related KEGG pathways enriched by DEPs (top 13). Data were analyzed by unpaired *t*-test and represented as means ± SD. * *p* < 0.05 and *** *p* < 0.001.

To characterize the transport properties of two different Cd exposures in the intestine of piglets, we then performed a GO and KEGG enrichment analysis on these DEGs (Figure 4G–I and Figure S3A). First, our main focus was to screen out those affected genes encoding transporters that may be involved in Cd transport. In regard to the KEGG pathway analysis, a total of 12 DEGs with the potential role of transporting Cd were co-enriched across all groups, among which 8 DEGs (*ZIP4*, *MT1a*, *TF*, *VDR*, *CTR1*, *SGLT1*, *SLC6A14*, *SLC26A4*) were enriched in the mineral absorption pathway (Figure 4C) and 5 DEGs (*ZIP4*, *ZIP1*, *ZIP6*, and *ZIP11*, *ZNT2*) were related to the transportation of zinc (Figure 4D). *ZIP4*, *ZIP1*, *ZIP6*, and *ZIP11*, aremembers of ZIP family, which were differentially expressed after Cd exposure, but their underlying contribution to Cd transportation is still unclear. Additionally, the involvement of transferrin (*TF*), the copper transport protein 1 (*CTR1*)*,* and the sodium-glucose cotransporter 1 (*SGLT1*) in Cd transportation have been reported (Figure 4C), while vitamin D receptor (*VDR*) and *MT1a* might be associated with the sensing and chelating Cd intracellularly (Figure 4F).

To explore whether and how Cd exposure affects the host metabolism, we performed GO and KEGG enrichment on the acquired DEGs with particular interest in their potential effects on metabolic functions. Our results suggested that Cd highly altered a wide variety of metabolic pathways in the duodenum of piglets. Specifically, in the CdCl_2_ vs. Ctrl group, the KEGG pathway analysis showed that the DEGs were enriched in fat digestion and absorption, cholesterol metabolism, metabolism of xenobiotics by cytochrome P450, retinol metabolism, metabolic pathways, and insulin resistance pathways (Figure 4G). In the KEGG enrichment of the Cdcr vs. Ctrl group, the top 13 pathways were identified, including mineral absorption, protein digestion and absorption, cholesterol metabolism, insulin secretion, retinol metabolism, fat digestion and absorption, and metabolic pathways (Figure 4H). Together, through these results we found that two Cd treatments (CdCl_2_ and Cdcr) had interesting common metabolic effects after exposure to piglets, they both had an impact on fat digestion and absorption (Figure S3B), and the cholesterol metabolism (Figure S3C), metabolic pathways, retinol metabolism, and insulin-related pathways were co-enriched across the two Cd treatments (CdCl_2_ and Cdcr) (Figure 4G–H). Moreover, CdCl_2_ and Cdcr also had different contributions to metabolic changes (Figure 4I), such as protein digestion and absorption (Figure S3D), retinol metabolism, metabolic pathways, and cholesterol metabolism, while several amino acid metabolism pathways exhibited differential enrichment (Figure 4I). Unexpectedly, we also found that chemical carcinogenesis, insulin secretion, maturity onset diabetes of the young, and apoptosis-multiple species were significantly enriched in the CdCl_2_ vs. Cdcr group, indicating that Cdcr may be more harmful than CdCl2, especially for the induction of cancer and diabetes.

Overall, the results illustrate that the intake of CdCl_2_ and Cdcr diets remarkably shapes the transcriptomic profile of piglets. Meanwhile, with the same criteria, the CdCl_2_ vs. Ctrl group had a fairly smaller number of DEGs (n = 77) than the Cdcr vs. Ctrl group (n = 1284), indicating a more remarkable alteration of the intestinal metabolic function in the piglets after fed Cdcr contained diet Meanwhile, the DEGs between the CdCl_2_ and Cdcr groups illustrate that the intake of CdCl_2_ and Cdcr diets both widely affect the metabolic function of piglets. However, these metabolic effects induced by CdCl_2_ and Cdcr are quite different, and the potential toxicity of Cdcr may be more intense than that of CdCl_2_ alone. These underlying mechanisms are still unavailable and require further investigation.

### 2.5. Proteome Analysis on Responses of the Duodenum of Piglets Fed Cd Containing Diets

To further elucidate protein expression events after Cd exposure in the duodenum of piglets, we utilized the TMT-labeled quantitative proteomics to quantify the proteome in our study. A total of 7753 proteins were identified in the whole intestinal samples; of these, 6865 proteins contained quantitative information. Next, we applied a cutoff of FC > 1.5 (up-regulated), FC < 1/1.5 (down-regulated), and *t*-test *p*-value < 0.05 as screening criteria among the three treatment groups. Subsequently, we gained a total of 87 (36 up-regulated and 51 down-regulated), 154 (79 up-regulated and 75 down-regulated), and 131 (54 up-regulated and 77 down-regulated) differentially expressed proteins (DEPs) in the CdCl_2_ vs. Ctrl, Cdcr vs. Ctrl, and Cdcr vs. CdCl_2_ groups (Figure 5A), respectively.

The KEGG and GO database was then utilized to map these DEPs onto pathways associated with Cd transportation, including the mineral absorption pathway, calcium ion transport pathway, divalent metal ion transport pathway, and divalent inorganic cation transport pathway. Of note, a total of 4 DEPs were mapped in the mineral absorption pathway (Figure 5B), including CLCA1, SGLT1, SLC6A19, and Na^+^/Ca^2+^ exchanger 1 (NCX1) (Figure 5C), which might be associated with Cd transportation in the duodenum of piglets. Furthermore, to determine the metabolism functions of the DEPs upon different Cd exposures, we conducted the GO and KEGG enrichment analyses of all DEPs in each groups (Figure 5D–F and Figure S4A–C).

Firstly, in the CdCl_2_ vs. Ctrl treatment group, GO enrichment exhibited that the up-regulated proteins were enriched for seven biological processes, including multiple fatty acid binding pathways (Figure S4A). Among the down-regulated proteins, 17 biological processes were enriched, including oxygen transport and malate metabolism (Figure S4A). In regard to the KEGG pathway analysis, among the top 14 significant pathways (Figure 5D), carbohydrate digestion and absorption, mineral absorption, and protein digestion and absorption pathways were enriched among the up-regulated proteins (Figure 5D), whereas six pathways, including lipolysis in adipocytes, pyruvate metabolism, retinol metabolism, and carbon metabolism, were significantly enriched among the down-regulated proteins (Figure 5D). Likewise, in the Cdcr vs. Ctrl group, GO enrichment revealed that up-regulated DEPs were significantly enriched in the glucose, amino acid digestion and transportation (Figure S4B), while the malic enzyme activity was reduced (Figure S4B). Subsequently, KEGG enrichment exhibited several proteins, fat, carbohydrate digestion and absorption pathways being up-regulated (Figure 5E), while the regulation of lipolysis in adipocytes, retinol metabolism, and pyruvate metabolism was down-regulated (Figure 5E). Notably, in our study, the co-enriched significant KEGG pathways for the up-regulated DEPs were carbohydrate digestion and absorption (Figure S4E) and protein digestion (Figure S4F). Additionally, we compared the effects of the Cdcr and CdCl_2_ exposure in the duodenum of piglets. The differences effects of feeding two Cd-contaminated rice on piglets were reflected in the GO enrichment analysis, including the up-regulated multiply peptidase activity, proteolysis, steroid metabolic process, calcium ion transport, divalent metal ion transport, and divalent inorganic cation transport pathways (Figure S4C), as well as the down-regulated several fatty acid bindings and immune responses. Meanwhile, KEGG enrichment exhibited the up-regulated protein digestion and absorption, type I diabetes mellitus, chemical carcinogenesis, cytochrome P450 related pathway, and Glutathione metabolism (Figure 5F), while revealing the down-regulated IL-17 signaling pathway, viral myocarditis, and bacterial invasion of epithelial cells (Figure 5F). Like transcriptomics, proteomics also showed that the Cdcr diet significantly enhanced cancer and type I diabetes mellitus pathways compared with CdCl2.

Collectively, the acquired data from the proteome were consistent with the transcriptomic: there were considerably different numbers of identified DEGs among all the groups, as the number of DEPs in the CdCl_2_ vs. Ctrl group was less than those in the others (Figure 5A). Furthermore, we found that the Cdcr diet had the unique capability of increasing the divalent metal ion transport based on the GO enrichment analysis (Figure S4B). Interestingly, in our results, both Cd-treatment groups up-regulated carbohydrate and protein digestion and absorption pathways (Figure S4E,F) although fewer metabolic functions were downregulated.

### 2.6. Integrative Analysis of the Transcriptome and Proteome Profiles

Our results also indicate that there are numerous biological differences between CdCl_2_ and Cdcr diets. We found that the Cdcr diet caused higher DEG/DEP amounts compared with the Ctrl group at the transcription and protein levels. To further analyze the impacts of Cd exposure in the intestines of piglets, we examined the crosstalk relationship in regard to the quantification of proteome and transcriptome. In the compared dataset of proteome and transcriptome, 5047 proteins or transcripts were identified both in proteome and transcriptome research (Figure S5A), and the expression of proteins and transcripts had a positive relationship, although this relationship was not strong (Figure S5B) [47].

**Figure 6:**
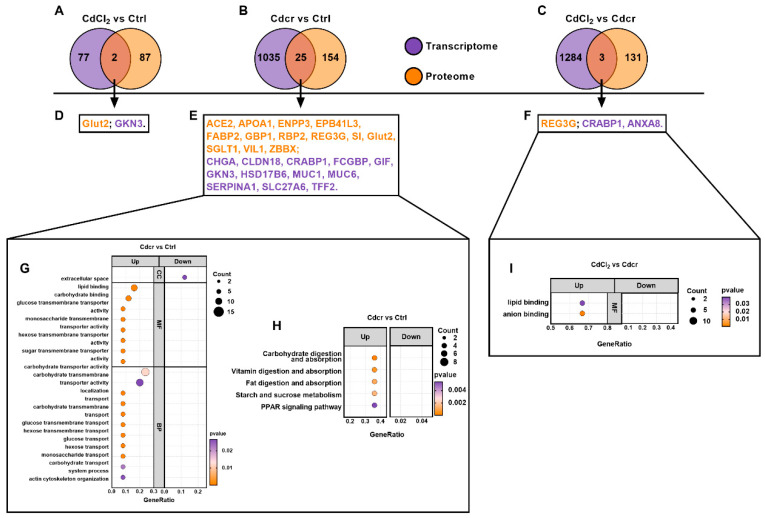
Integrative Analysis of the Transcriptome and Proteome after piglets were fed with CdCl_2_ and Cdcr contained diet. (**A**–**C**) Transcripts or proteins were both identified in the proteome and transcriptome in each group. (**D**–**F**) Information on transcripts or proteins. All GO (**G**) and KEGG (**H**) pathways were enriched with transcripts or/and proteins in the Cdcr vs. Ctrl group. (**I**) All GO pathways were enriched with transcripts or/and proteins in the CdCl_2_ vs. Cdcr group.

In these targets, we gained two, 25, and three matched synchronously regulated transcripts or proteins in the CdCl_2_ vs. Ctrl group, Cdcr vs. Ctrl group, and the Cdcr vs. CdCl_2_ group, respectively (Figure 6A–C). Among the results, Glut2 was synchronously regulated, which might be related to Cd transportation (Figure 6D). Next, we conducted the GO and KEGG enrichment analyses of transcripts or proteins in the Cdcr vs. Ctrl group and the CdCl_2_ vs. Cdcr group. As for Cdcr vs. Ctrl, 19 of 20 pathways were up-regulated in the GO enrichment, including multiple carbohydrate and glucose metabolism pathways (Figure 6E,G). In the KEGG enrichment, all five pathways were up-regulated, including carbohydrate digestion and absorption, vitamin digestion and absorption, fat digestion and absorption, and starch and sucrose metabolism (Figure 6E,H). For the CdCl_2_ vs. Cdcr group, three transcripts or proteins enriched in lipid and anion binding were up-regulated in the context of GO enrichment analysis (Figure 6F,I). Taken together, we found that the Cdcr diet had the most obvious impacts on the duodenum of piglets, especially for metabolism-related pathways. It is more noteworthy that chemical carcinogenesis and diabetes pathways were found to be significantly enriched in the Cdcr group than CdCl_2_ in both the transcriptome and metabolome. Multi-omics results indicated that Cdcr exposure in the intestine had a greater impact than CdCl_2_, and had a greater effect on rewiring the metabolism and pathogenic pathways.

## 3. Discussion

Cd pollution is a serious environmental security issue across the world, and Cd exposure from contaminated food seriously threatens the health of the body [8]. In our results, we showed that feeding with both types of Cd-contaminated rice significantly induced intestinal absorption (especially duodenum and proximal jejunum) and promoted circulating Cd concentration. Studies on potential transporters of Cd have found that DMT1, ZIP8 and TRPV6 DMT1, ZIP8 and TRPV6 were significantly increased in both Cd exposure groups, and these transporters were more highly expressed in most segments in the CdCl2 group. Meanwhile, the CdCl_2_ group had more Cd content in the intestine, liver and kidneys, but less in blood circulation, which might be the reason why the intestinal pH of CdCl_2_ was in the optimal range for DMT1, ZIP8 and/or TRPV6 transport efficiency [48]. Furthermore, the lower pH may favor the secretion of Cd (basolateral to apical) to reduce Cd accumulation in the epithelium, rendering it a potential strategy to alleviate Cd exposure [34,49]. Furthermore, we found that FPN1 significantly decreased in both Cd exposure groups, and its expression in CdCl_2_ was lower than that in the CdCr group, which was responsible for retaining Cd in intestinal epithelial cells and preventing the expansion of toxicity from entering the systemic circulation [50]. Furthermore, the expression of MT3 was decreased in the CdCl_2_ and Cdcr groups, while the expression of MRP1 was increased. We proposed that the compensative increase in expression of MRP1 enhanced the scavenging of intracellular Cd by expelling Cd from the cell. Despite reduced MT expression, studies suggest its expression is independent of its metal sequestering efficiency [51], even if it is down-regulated [52,53], but further study is still needed. These results indicate that Cd contaminants have different tissue distribution and deposition rates, which might be related to the involvement of transport carriers in the intestine. However, in our study, only intestinal absorption differences were shown, and Cd deposition in different organs may also vary and be related to transporters, requiring a deeper understanding.

Furthermore, we quantified 12 DEGs and four DEPs associated with Cd absorption and transportation in the intestine using RNA-seq and proteomic techniques. ZIP family members are the most abundant transporters from omics that may participate in Cd absorption, such as ZIP1, ZIP4, ZIP6, ZIP-11, and ZnT-2. However, there is no relevant research demonstrating that these ZIP family transporters are involved in Cd transport, so a detailed characterization of these targets is needed. Next, some transporters have been reported to be involved in Cd transport, including TF, CTR1, SGLT1, and NCX1. TF is markedly decreased in the Cdcr group, which may be associated with liver damage and be a precursor of anemia. Although this study did not find a decrease in Fe levels in the blood, there is research indicating that Cd competes with iron for binding to TF. As the concentration of Cd increases, Cd binds to TF at lower affinity sites via electrostatic force and releases Fe [54]. This free Fe further induces organ damage [55,56], such as in the liver, where TF is synthesized, eventually resulting in the decrease in TF production [55]. The expression of the CTR1 was decreased after Cdcr diet exposure, which was congruent with the data previously described in the literature using a zebrafish model [57]. During Cd exposure, cells attempted to retain CTR1 in the Golgi apparatus to reduce its retention on the cell membrane to cut off the uptake of Cd and cytotoxicity [58]. For SGLT1 and NCX1, there are only reports indicating that their transport function is inhibited by Cd, but whether they transport Cd is still unclear [59,60,61,62,63,64]. In addition to MT, which has been described above, we found that several proteins, such as MT1A, and VDR, also participated in the Cd detoxification. In our results, the expression of VDR increased in both Cd-treated groups, and cytochrome P450-related pathways were also significantly enriched in KEGG. VDR is a member of the steroid hormone receptor family, whose downstream targets are involved in mineral metabolism. It has been widely reported that the activation of VDR is associated with apoptosis, with a striking example being Cd-induced apoptosis being antagonized by the activated VDR/CREB1 pathways in the spleens of pigs [65]. Cd exposure has been shown to interfere with the activity of cytochrome P450 enzymes [66], such as VD3 metabolic enzymes CYP27B1 and CYP24A1 [67,68], accompanied by low serum Vitamin D3 and Ca levels. Therefore, the expression of VDR in our study may be a compensatory increase after Cd exposure, and it has been proven that Cd toxicity can be offset by supplementing vitamin D3 and Ca [69,70].

Of note, in our study, we demonstrated that Cd exposure significantly induces wide alterations in piglet metabolism by the results of RNA-seq and proteome. Consistent with previous studies [71], KEGG analysis identified that carbohydrate digestion and absorption, fat digestion and absorption, cholesterol metabolism, and protein digestion and absorption pathways were significantly affected by Cd exposure.

For the carbohydrate metabolism, the CdCl_2_ and Cdcr exhibited a differently enriched diabetes pathway. In vitro and in vivo studies have shown that Cd exposure interferes with the expression of metabolic pathways and related enzymes in glycolysis [72,73], glutamine [73], and the TCA cycle [74]. Meanwhile, a longitudinal prospective study also showed that Cd exposure significantly increased fasting glucose and T2DM prevalence [75]. Interestingly, studies have found that Cd promotes glycolysis at the beginning of the experiment. However, long-term exposure to Cd in turn inhibits glycolysis, and Cd exposure is associated with prediabetes and diabetes, both of which are induced in a concentration-dependent manner [76,77]. These findings reveal that metabolic remodeling during Cd exposure and maintaining a stable metabolism are critical, and metabolites, such as Zn [36], vitamin C [78], and calcium [79] have been shown to have the potential to mitigate Cd toxicity.

For the relationship between Cd and fat, studies show that Cd has a fat accumulation effect [70], especially in males [80], implying that Cd exposure may instead increase body weight to cover its hazards. Notably, in our results, Cd exposure increased the enrichment of steroid hormone-related pathways in transcriptomics. Research has defined Cd as a metal hormone [81] which can interact with the estrogen receptor alpha (ERα) to translocate into the nucleus, which increases the threat of metabolic disorders. Therefore, the metabolic effects of Cd exposure on males and females may have different underlying mechanisms. Furthermore, previous research has shown that Cd exposure induces cholesterol redistribution by up-regulating ATP-binding cassette (ABC) transporters and down-regulating oxysterol-binding proteins (OSBPs) [82]. In addition, the serum levels of total cholesterol, LDL-C, and non-HDL-C are elevated in humans, mice, and rats after Cd exposure [83,84,85]. In our results, *ABC-G2* was up-regulated in proteome and *OSBP2* was down-regulated in RNA-seq, suggesting that Cd promotes the redistribution of cholesterol from tissue to serum [82].

Importantly, Cd exposure also alters protein metabolism. In our results, the activity of carboxypeptidase increased with the enrichment of Cdcr groups. Cd exposure was reported to reduce the activity of dipeptidase and Na^+^/K^+^ ATPase and to constrain carboxypeptidase A in a concentration-dependent manner [86], which has influences on the absorption of proteins in the proximal small intestine [87] and pancreas [86].

In summary, we found that Cdcr is significantly different from CdCl_2_ in terms of absorption, transport, in vivo distribution, metabolic rewiring and pathogenicity (like cancer and diabetes) after analysis at the organ Cd deposition, transcriptomic, and/or proteomic levels. Importantly, we propose that experimentally simulated Cd exposure (CdCl_2_) may have limitations in reflecting environmental Cd contamination. In addition, the Cd in contaminated rice may also be determined by the growth environment and source of pollution, which requires further study. We hope that our study can provide new perspectives on the biological characteristics of Cd pollutants and advance in-depth research on the biological toxicity of Cd. Therefore, it is necessary to further dissect the unique hazards that different Cd compounds and even natural Cd pollutants may have in order to promote more in-depth and systematic research on Cd poisoning.

## 4. Materials and Methods

### 4.1. Chemical Reagents

Cd contaminated rice (Supplied by the Institution of Subtropical Agricultural, Chinese Academy of Sciences), CdCl_2_ (TCI, China), Trizol (Invitrogen, Carlsbad, CA, USA), DEPC (Sigma, St. Louis, MA, USA), Isopropanol (Sinopharm 40049961, Shanghai, China), Chloroform (Sinopharm 10006818, China), Ethanol (Sinopharm 40021279, China), RIPA (Wellbio, China), BCA protein assay kit (Wellbio, China), SDS (Sigma, L3771, USA), HNO_3_ (Sigma, 80089260, USA), H_2_O_2_ (Sinopharm 10011208, China), Cd standard solution (Sinopharm 53205961, China), Fe standard solution (Sinopharm 53205160, China), Zn standard solution (Sinopharm 10006818, China), Cu standard solution (Sinopharm 53205160, China), Mn standard solution (Sinopharm 53204660, China). 

### 4.2. Animals and Cd Exposure

A total of 24 healthy male weaned piglets (Large White × Landrace × Duroc, 35-day-old, weighted at 10.0  ±  1.0 kg) were selected and randomly allocated into three groups. Piglets were housed under the Institute of Subtropical Agriculture Chinese Academy of Sciences. Overall, piglets in the three groups were given a rice contained diet (Ctrl), a CdCl_2_-added rice diet (CdCl_2_), and a Cd-contaminated rice diet (Cdcr). The diets were based on growing-finishing pigs’ nutritional requirements (NRC, 2012) in order to construct a rice-soybean-based diet, and the composition and nutrient levels are shown in Table 1.

For the rice in Ctrl group, the Cd content was 0.25 ± 0.01 mg/kg in uncontaminated rice and 0.15 ± 0.01 mg/ kg after the formulated diet (contained 61.59% rice, Table 1), fed to the piglets in the Ctrl group. For the Cd-contaminated rice, it was purchased from Cd-contaminated areas. After detection, the Cd content in rice was 1.87 ± 0.29 mg/ kg and 1.15 ± 0.04 mg/ kg after the formulated diet (contained 61.59% rice, Table 1), fed to the piglets in the Cdcr group. Finally, we added 1.32 mg/ kg CdCl_2_ into the uncontaminated rice to equilibrate the Cd level to those in the Cdcr group, after the formulated diet, fed to the piglets in the CdCl_2_ group.

To demonstrate that dietary Cd is absorbed through the intestines and enters the blood circulation, on day 14, the anterior vena cava blood was collected at the intersection of the two forelimbs and the trachea, and then the piglets from each treatment group were anesthetized and euthanized. The abdominal cavity was quickly opened and hepatic portal venous blood and mesenteric venous blood samples were collected. Subsequently, samples from liver, kidney, and small intestine segments were collected and then snap-frozen in liquid nitrogen and stored at −80 °C until further analysis. The contents in stomach and different segments of the small intestine were collected into centrifuge tubes, and the pH levels were measured.

### 4.3. Metal Elements Determination

Metal elements (Cd, Fe, Cu, Zn, Mn) concentrations in the samples were quantified using an inductively coupled plasma emission spectrometer (ICP 5110, Agilent, Santa Clara, CA, USA) according to the methods of the National Standards of the People’s Republic of China. This test was conducted according to the previous method of our laboratory, like Dong et al. [88]. The detection method is as follows: tissue samples were weighed (0.5000 g) in Teflon crucibles and digested using a combination of 7 mL of 65% HNO_3_ and 1 mL of 30% H_2_O_2_ in a microwave digestion system (Milestone-Start D Microwave laboratory system, Italy). After complete evaporation, the samples were dissolved in 1% HNO_3_ and submitted to the ICP analyses. All samples were analyzed in duplicate. A standard curve was constructed for the determination of the metal element concentrations by using a serially diluted standard solution and 1% HNO_3_ as a blank.

### 4.4. Western Blots

Immunoblotting analysis was performed following the standard procedures and according to the previous method [89]. Briefly, 0.025 g small intestine samples were ground and lysed in ice-cold 200 μL radioimmunoprecipitation assay (RIPA, Wellbio, China) lysis buffer for 10 min. The supernatant was used to measure the protein concentration using a bicinchoninic acid (BCA) protein assay kit (Wellbio, China). After separating by 10% sodium dodecyl sulfate (SDS, Sigma, USA) -polyacrylamide gel electrophoresis (PAGE) electrophoresis (Bio-rad, Hercules, CA, USA), proteins were transferred onto a polyvinylidene fluoride (PVDF) membrane (BioRad, Hercules, CA, USA), and blocked with 5% bull serum albumin (BSA, Sigma, USA) Tris-Tween-buffered saline buffer (TBST, Sigma, USA) for 1 h. Then, the membranes were incubated with the primary antibodies (Table 2) overnight at 4 °C. Subsequently, the HRP-conjugated secondary antibodies (anti-rabbit 1:6000, Proteintech; anti-mouse 1:5000, proteintech.) were incubated for 90 min at 37 °C. Finally, the membrane-developed blots were analyzed using a light imaging system (Thermo, Waltham, MA, USA). The signal density of the film was viewed and analyzed using AlphaImager 2200 software. The information antibody is shown in Table 2.

### 4.5. RNA Sequencing and Data Processing

All of these methods were referred to in a previous study [90]. Briefly, the total RNA was extracted from the duodenum of the piglets, and Qubit(Thermo, Waltham, MA, USA) and Agilent 2100 (Santa Clara, CA, USA) were used to detect the concentration and integrity of the amplified products. First, magnetic beads with Oligo(dT) were used to enrich the mRNA, then add Fragmentation buffer fragmented mRNA, use the fragmented mRNA as a template, and use six-base random primers (random hexamers) to synthesize the first cDNA strand. Then buffer, dNTPs, RNase H, and DNA polymerase I (Sigma, USA) were added to synthesize the second cDNA strand. After purification with magnetic beads and elution with EB buffer, end repair was performed and sequencing adapters are connected. Then, magnetic beads were used for fragment size selection, and, finally, PCR amplification was performed and the built transcriptome library was used for on-machine sequencing. Clean data were obtained by filtering low-quality reads from raw data and mapping them to the reference genome. The edgeR (v3.16.5) package was used to select differentially expressed genes from the transcriptome (RNA-seq) (FC ≥ 2, FDR ≤ 0.01). The enrichment analysis of the different express genes (DEGs) was performed with topGO, KEGG and Kobas.

### 4.6. Proteomics and Data Analysis

All of these methods were referred to in a previous study [91]. Briefly, fresh duodenum segments isolated from piglets were lysed for protein extraction. After determining the concentration, samples were labeled by the Tandem Mass Tag (TMT) labeling kit. Proteomic analyses were performed on a Q Exactive Plus mass spectrometer (Thermo Fisher Scientific, Waltham, MA, USA). The resulting MS/MS data were processed using the Maxquant search engine (v.1.5.2.8). Tandem mass spectra were searched against the Sus Scrofa database (40,710 sequences) and concatenated with the reverse decoy database. FDR was adjusted to <1%, and the minimum score for peptides was set to >40.

### 4.7. Statistical Analysis

All statistical analyses were performed using Prism 9.0 software (GraphPad), and the results are represented as means ± SEM or SD. Data between the two groups were analyzed by unpaired *t*-tests (Welch’s correction) and Mann–Whitney U-tests. Differences with *p* < 0.05 were considered significant.

## Figures and Tables

**Table 1:** Composition and nutrition levels of experimental diets (%, DM basis).

Ingredients	Ctrl Diet (%)	CdCr Diet (%)
Brown rice	61.59	
Soybean (43%)	12.61	
Extruded soybean	10.00	
Whey powder (3%)	4.00	
Fish meal	4.00	
Soybean oil	2.17	
Bran	1.50	
Premix	1.00	
Limestone	0.96	
CaHPO4	0.49	
Lysine (98%)	0.47	
ZnO	0.30	
Feed acidifier	0.30	
Threonine	0.17	
Methionine	0.15	
NaCl	0.10	
Mold inhibitor	0.10	
Choline chloride	0.05	
Antioxidant	0.05	
Nutrient levels		
Crude protein	19.00	
Calcium (Ca)	0.70	
Available phosphorus	0.35	
Lysine	1.40	
Methionine + Cysteine	0.79	
Threonine	0.87	
Tryptophan	0.21	
Digestive energy, Mcal/kg	3.49	
Cadmium (Cd)	0.15 mg/kg *	1.15 mg/kg *

Notes: The Cd in this diet derived from the brown rice. The Cd content in rice of the Ctrl group ingredients is 0.25 ± 0.01 mg/kg; in the rice of CdCr group ingredients is 1.87 ± 0.29 mg/kg. * Calculated values.

**Table 2:** The antibodies used in the present study.

Antibody Name	Origin	Dilution Ratio	Source	Identifier
DMT1	Rabbit	1:1000	Abcam	ab133402
FPN1	Rabbit	1 µg/mL	Abcam	ab58695
ZIP8	Rabbit	1:500	Proteintech	20459-1-AP
MT3	Rabbit	1:500	Proteintech	12179-1-AP
TRPV6	Rabbit	1:500	Proteintech	13411-1-AP
MRP1	Mouse	1:500	Abcam	ab32574
β-actin	Mouse	1:5000	Proteintech	60008-1-Ig

## Data Availability

Data contained within the article. The original contributions presented in the study are included in the article/Supplementary Materials, further inquiries can be directed to the corresponding author/s.

## Supplementary Materials

The following supporting information can be downloaded at: https://www.mdpi.com/article/10.3390/ijms25126474/s1.

## Abbreviations

MT	metallothionein
DMT1	divalent metal transporter 1
FPN1	ferroportin 1
ZIP	Zrt-/Irt-like protein
TRPV6	Transient receptor potential cation channel subfamily V member 6
MRP1	multidrug resistance protein 1
TF	transferrin
CTR1	copper transport protein 1
SGLT1	sodium glucose cotransporter 1
VDR	vitamin D receptor
NCX1	Na^+^/Ca^2+^ exchanger 1
