# Supplementary material for: Integrating Transcriptomics and Proteomics to Characterize the Intestinal Responses to Cadmium Exposure Using a Piglet Model
Source: Int J Mol Sci. 2024 Jun 12;25(12):6474. doi: 10.3390/ijms25126474 (PMC11203886; doi:10.3390/ijms25126474)
Supplement: Supplementary file 1 [file ijms-25-06474-s001.zip › ijms-2993991-supplementary.pdf]

Integrating transcriptomics and proteomics to characterize the intestinal response to cadmium exposure using a piglet model

Yikun Li[1,2], Yiling Pan[2], Yulong Yin [2,*], Ruilin Huang [2,*]

Table of contents

Supplemental figure 1..2

Supplemental figure 2..3

Supplemental figure 3..4

Supplemental figure 4..5

Supplemental figure 5..6

Supplemental Figures

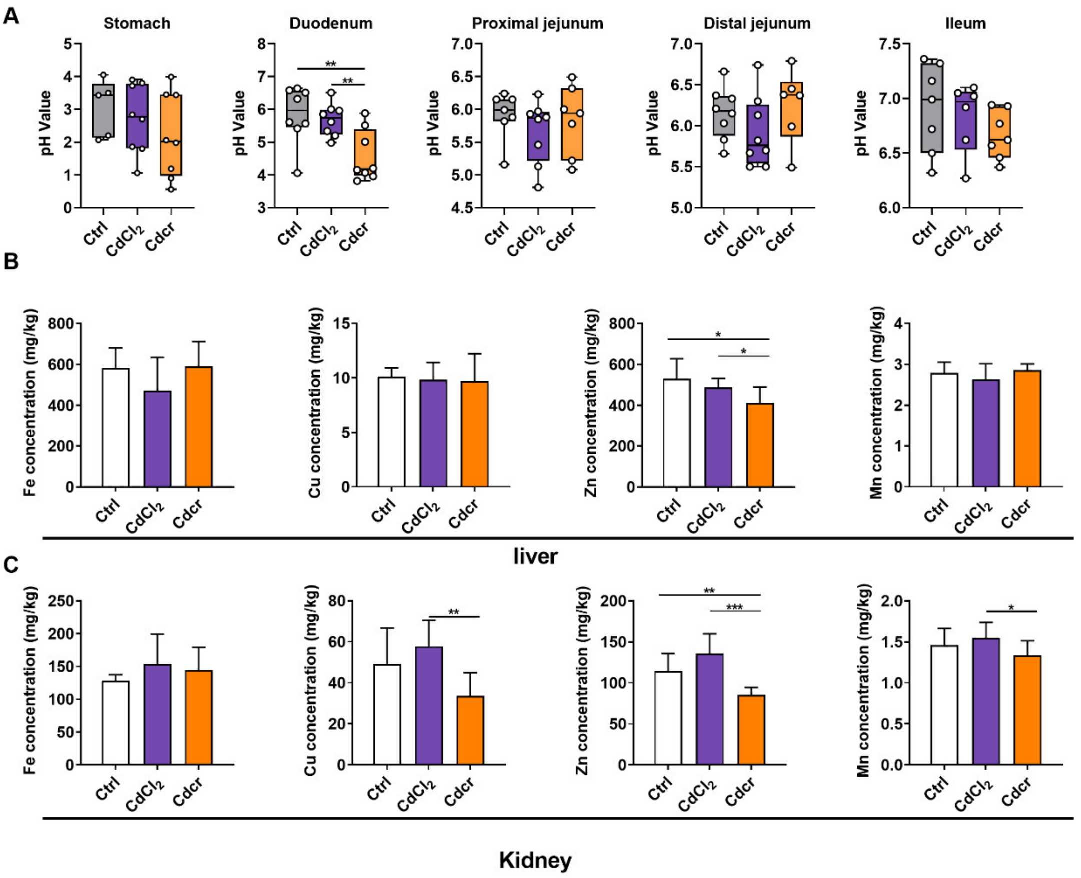

Figure S1. Value of the gastrointestinal pH and liver and kidney metal deposition of piglets fed $CdCl_2$ and/or Cdcr containing diets. (A) pH value in the gastrointestinal tract of piglets after exposure to $CdCl_2$ and Cdcr (n = 8) (represented as means ± SEM). (B) Changes in Fe, Cu, Zn and Mn contents in the liver and kidney of piglets after exposure to $CdCl_2$ and Cdcr (n = 8). Data were analyzed by unpaired *t*-test and means ± SD. *P < 0.05, **P < 0.01 and ***P < 0.001.

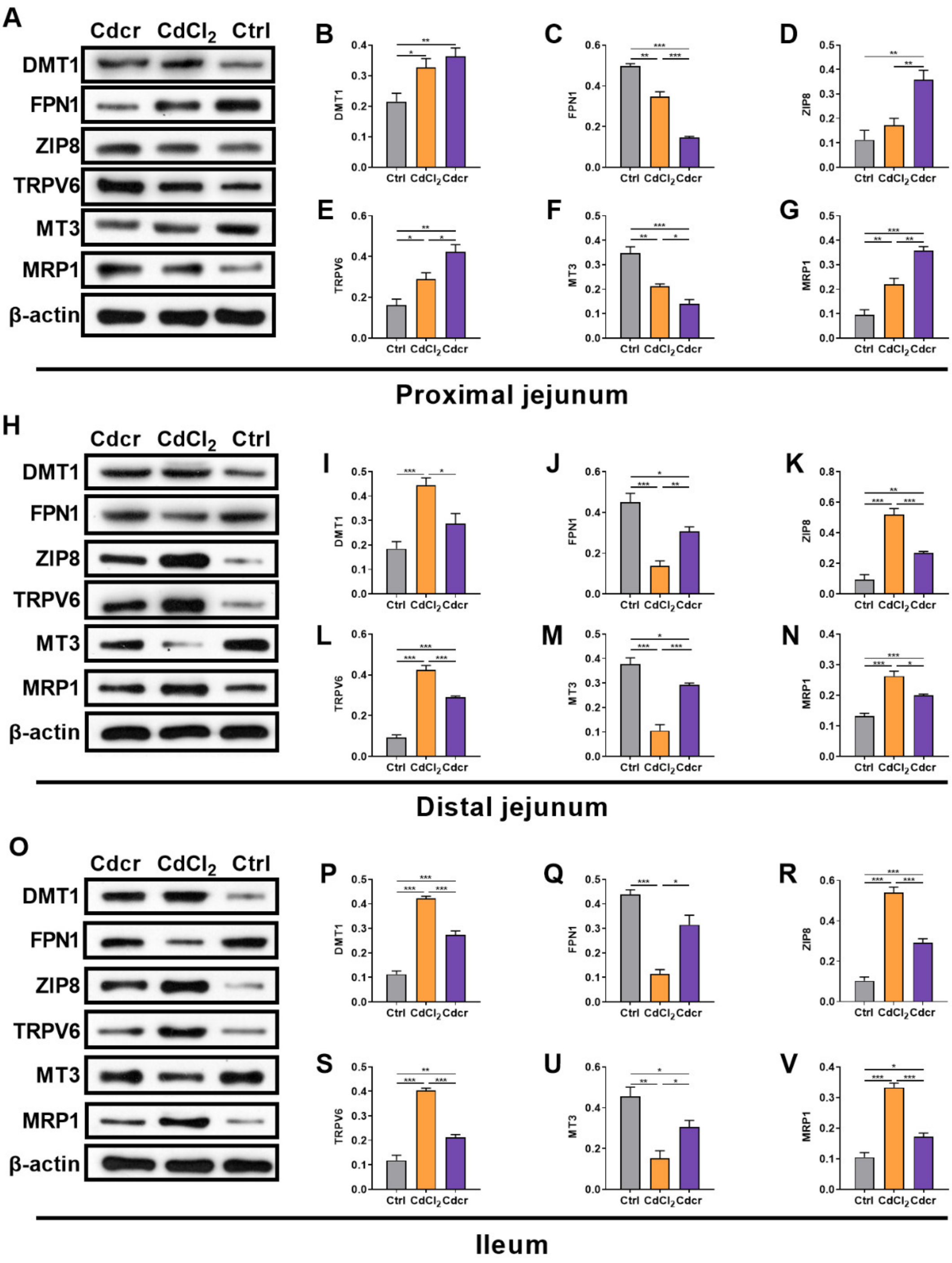

Figure S2. Expression of Cd-related transporters in the jejunum and ileum of Cd-exposed pigle. Protein expression of DMT1, FPN1, ZIP8, TRPV6, MT3 and MRP1 in the proximal jejunum**(**A-G), distal jejunum(H-N) and ileum(O-V) of Cd exposed piglets (n = 4). Data were analyzed by unpaired *t*-test and represented as means ± SD. *P < 0.05, **P < 0.01 and ***P < 0.001.

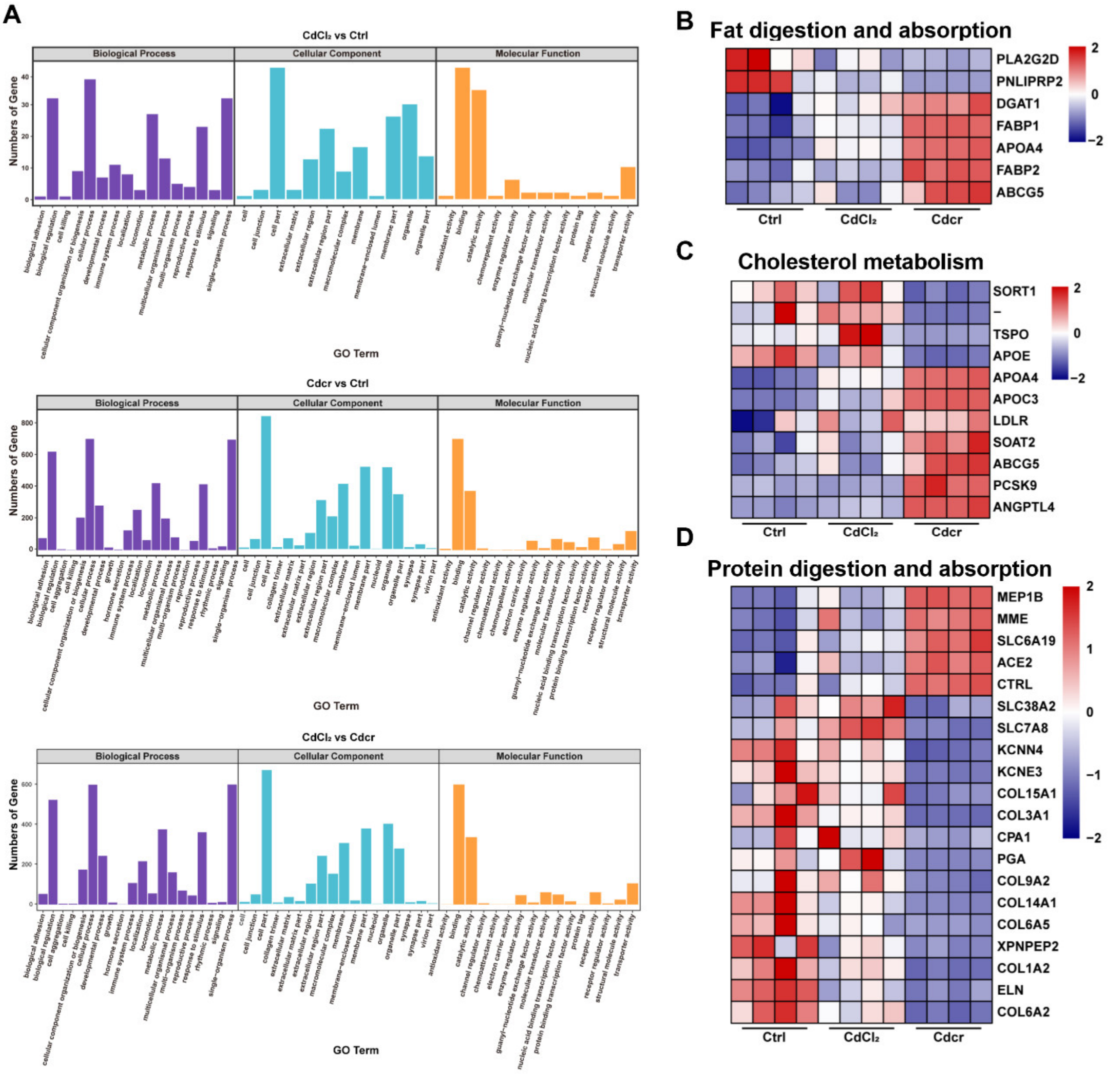

Figure S3. Transcriptomic analysis on responses of the duodenum of piglets fed Cd containing diets (A) GO enrichment of DEGs. Heatmaps demonstrated DEGs in Fat digestion and absorption (B), cholesterol metabolism (C), protein digestion and absorption (D) pathways.

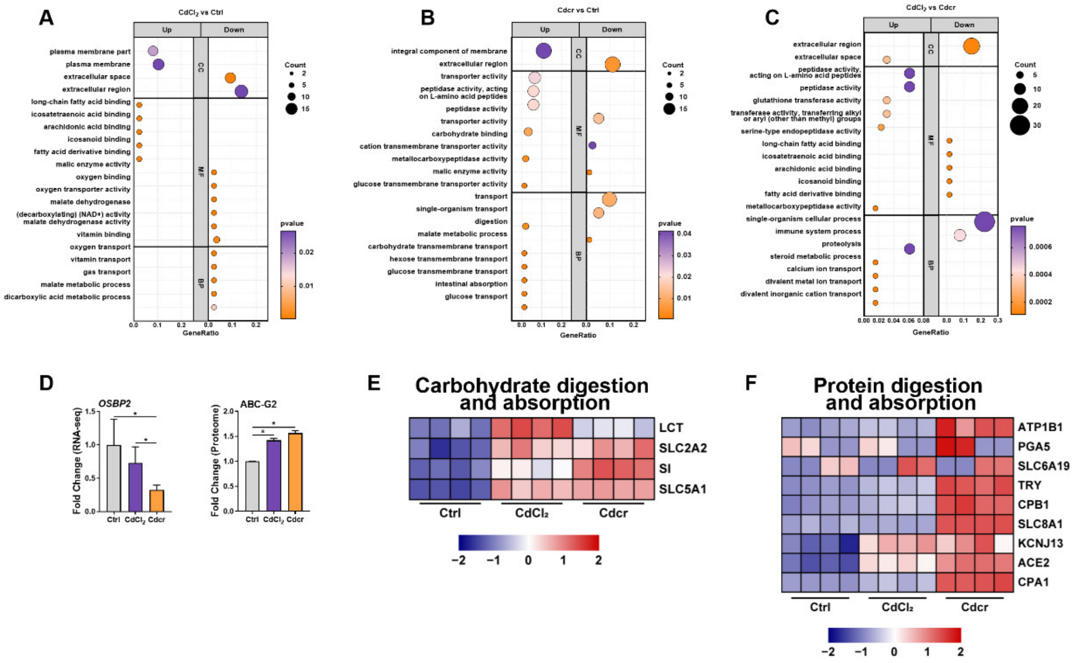

Figure S4. Proteome analysis on responses of the duodenum of piglets fed Cd containing diets. (A-C) GO enrichment of DEGs. CC: cellular components, BP: biological processe, MF: molecular function. Heat map demonstrated DEGs in carbohydrate digestion and absorption (E), and protein digestion and absorption pathways(F).

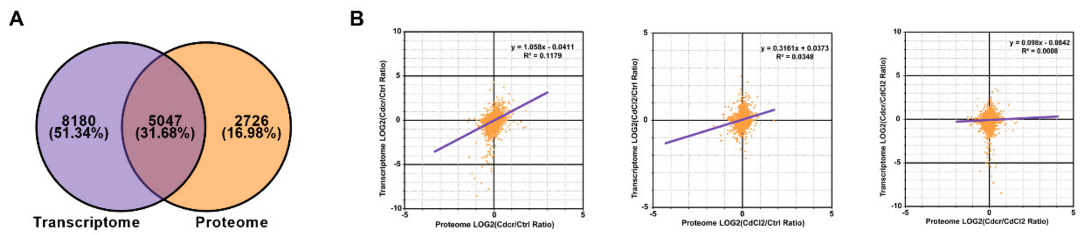

Figure S5. Transcriptome and Proteome Crosstalk Analysis. (A) Transcriptome and Proteome identified compared Venn diagram. (B) Transcriptome and Proteome quantification relationship.
